# Tissue-specific microbiota dictates the competitive dynamics of *listeria* species colonization

**DOI:** 10.1080/01652176.2026.2622742

**Published:** 2026-02-02

**Authors:** Juliette Poujol de Molliens, Carla Palacios-Gorba, Jazmin Meza-Torres, Jesús Gomis, Angel Gómez-Martín, Juan J. Quereda

**Affiliations:** a Research Group Listeria: Biology and Infection (LisBio). Departamento Producción y Sanidad Animal, Salud Pública Veterinaria y Ciencia y Tecnología de los Alimentos, Facultad de Veterinaria, Universidad Cardenal Herrera-CEU, CEU Universities, Alfara del Patriarca, Valencia, Spain; b Sir William Dunn School of Pathology, University of Oxford, Oxford, UK; c Research Group Microbiological Agents Associated with Animal Reproduction (ProVaginBIO). Departamento Producción y Sanidad Animal, Salud Pública Veterinaria y Ciencia y Tecnología de los Alimentos, Facultad de Veterinaria, Universidad Cardenal Herrera-CEU, CEU Universities, Alfara del Patriarca, Valencia, Spain

**Keywords:** Foodborne, reproductive, gut, pathogens, virulence, ecology, environment, ruminant

## Abstract

The genus *Listeria* is heterogeneous and contains pathogenic and nonpathogenic species. Pathogenic *L. monocytogenes* and *L. ivanovii* have different environmental distributions, infect different hosts, and cause distinct syndromes. Here, we evaluated whether responses of different *Listeria* species to diverse host niches contribute to virulence heterogeneity and influence their environmental distribution. We assessed resistance to gastric and intestinal fluids, gut and uterus microbiota, and semen. *L. monocytogenes* was more resistant than *L. ivanovii* in gastric fluid, whilst *L. seeligeri* and *L. valentina* showed an intermediate phenotype. All the tested *Listeria* species resisted the intestinal fluid. Gut microbial communities outcompeted and eliminated *L. ivanovii* and *L. valentina*. However, *L. monocytogenes* and *L. seeligeri* outcompeted intestinal commensal bacteria. Our findings suggest that, unlike *L. monocytogenes* and *L. seeligeri*, the tested *L. ivanovii* are unlikely to colonize the gastrointestinal tract of ruminants, which would reduce replication, fecal shedding, and environmental distribution. However, the ability of *L. ivanovii* to survive within uterine microbiota and semen suggests that the tested clones could persist in the urogenital tract of ruminants. Then venereal transmission could be more probable than the oral route, which could explain why *L. ivanovii* is associated with abortion outbreaks and not encephalitic cases.

## Introduction

The genus *Listeria* consists of 29 widely distributed bacterial species, among which only *Listeria monocytogenes* (*Lm*) and *Listeria ivanovii* are recognized as zoonotic pathogens (Brown et al. 2025). *Listeria* species are diverse and display high diversity in virulence and environmental distribution (Orsi and Wiedmann 2016; Hafner et al. 2021). Pathogenic species encode, among other virulence factors, the *Listeria* pathogenicity island 1 (LIPI-1) and the *inlA-inlB* locus, which are necessary for key steps of intracellular parasitism (e.g. host cell adhesion, internalization, intracellular survival, and dissemination). *Lm* is the primary cause of listeriosis, affecting both ruminants and humans, particularly, the elderly, pregnant women and immunocompromised individuals. In these hosts, *Lm* causes septicemia, meningoencephalitis, abortion, and stillbirth (Quereda et al. 2021). In contrast, *L. ivanovii*, which comprises two subspecies (*L. ivanovii* subsp. *ivanovii* (*Liv*) and *L. ivanovii* subsp. *londoniensis* (*Lond*)), is mainly associated with ruminants, causing fetoplacental infections in pregnant ewes, goats, and cows. Interestingly, *L. ivanovii* has never been associated with neurological infections (Sergeant et al. 1991; Alexander et al. 1992; Vázquez-Boland et al. 2001; Guillet et al. 2010; Rossi et al. 2022). Moreover, only eleven human cases of gastroenteritis and septicemia caused by *L. ivanovii* have been reported to date (Guillet et al. 2010; Mani et al. 2025). Outbreaks of listerial encephalitis in domestic ruminants are associated with silage contamination; however, the source of infection for listerial abortion outbreaks remains elusive. *Lm* population is classified into four distinct lineages, with lineage I being strongly associated with clinical cases and considered hypervirulent (Maury et al. 2016). Notably, lineage I is predominantly linked to ruminants. The frequency of *Lm* fecal carriage ranges between 3.8% and 60% in domestic ruminants (Esteban et al. 2009; Hurtado et al. 2017; Palacios-Gorba et al. 2021a). Furthermore, *Lm* and *Liv*/*Lond* differ in prevalence and isolation sources. *Lm* is more prevalent than *Liv*/*Lond* in soil and host-associated environments (Hafner et al. 2021). *Liv*/*Lond* prevalence in feces is generally inferior to 2% in all the sampled niches, with some notable exceptions in poultry and wild rodents (Gaya et al. 1996; Gwida et al. 2011; Alvarez-Ordóñez et al. 2015; Cao et al. 2019; Palacios-Gorba et al. 2021a, 2021b, 2023). A tendency for *L. ivanovii* to be more easily isolated from aborted fetuses, milk, udders, tonsils, and vaginal swabs—particularly from sheep—rather than in fecal samples has been observed in some cases (Alexander et al. 1992; Gaya et al. 1996; Palacios-Gorba et al. 2021b, 2023). Different authors have suggested that *L. ivanovii* may be a venereal pathogen in small ruminants rather than a foodborne pathogen (Gray 1963.; McDonald 1967; Smith et al. 1967; Alexander et al. 1992; Wiedmann et al. 1999).

Some *L. seeligeri* (*Lseel)* isolates also possess LIPI-1 (as *L. monocytogenes* and *L. ivanovii*) and show hemolytic capabilities. Since an isolated *L. seeligeri* human meningitis case has been reported (Rocourt et al. 1986), it remains to be elucidated if this species is pathogenic in a specific, yet to be revealed, host species (Orsi and Wiedmann 2016). *L. valentina* (*Lval*) is a recently discovered species isolated from the feces of healthy sheep. It does not possess pathogenicity islands or virulence factors and differentially to *Lm*, *Liv*, *Lond,* and *Lseel*, belongs to the *sensu lato* group, and does not grow at low temperatures (Quereda et al. 2020).

The acidity of the gastric juice is the first barrier against pathogens ingested with food or water (Smith 2003). After passing through the stomach and reaching the small intestine, ingested *Listeria* cells must survive bile, pancreatic enzymes, and high osmolarity conditions. The use of artificial gastrointestinal systems that replicate the dynamics of gastrointestinal transit provides valuable information about factors affecting the survival of *Listeria* in the gastrointestinal tract (Ramalheira et al. 2010). Once these barriers have been endured, gut commensal bacteria can directly protect against pathogen colonization by competing for nutrients or producing bacteriocins (Oliveira et al. 2025). Similarly, uterine microbiota might provide colonization resistance against venereal pathogens (Benner et al. 2018; Wang et al. 2021).

To the best of our knowledge, no previous study has focused on using *in vitro* and *ex vivo* gastrointestinal systems of gut and reproductive microbiota models to decipher *Listeria* species virulence heterogeneity. Here, to gain insights into the ecological preferences of *Listeria* species described in the previous paragraphs, we examined differences in gastrointestinal survival among *Lm*, *Liv*, *Lond*, *Lseel*, and *Lval* using an *in vitro* simulation model of the stomach and small intestine. Next, we investigated their survival in *ex vivo* gut and uterine microbiota models and in semen.

## Material and methods

### Bacterial strains and CFU enumeration

In all the experiments, we used two *L. ivanovii,* one *Liv* and one *Lond,* one *Lm* belonging to CC1 from lineage I, one *L. seel* and one *Lval,* a specie isolated for the first time by our group in the south-east of Spain (Quereda et al. 2020). Their genetic characteristics and isolation sources are detailed in Table S1.

Colonies were obtained from −80 °C frozen aliquots that were plated on BHI agar plates and grown at 37 °C. A colony was then inoculated into BHI and grown overnight at 37 °C at 250 rpm for all *Listeria* species to OD = 0.9–1.2.

### Simulated gastrointestinal fluids preparation

To prepare simulated gastrointestinal fluid (SGF) and simulated intestinal fluid (SIF), the following stock solutions were used: SGF consisted of sodium chloride (175.3 g/L), sodium dihydrogen phosphate (88.8 g/L), potassium chloride (89.6 g/L), calcium chloride (22.2 g/L), ammonium chloride (30.6 g/L), glucose (65.0 g/L), glucuronic acid (2.0 g/L), urea (25.0 g/L), glucosamine (33.0 g/L), bovine serum albumin (1.0 g/L), mucin type II from porcine stomach (3.0 g/L) and pepsin (1.3 g/L). The pH was adjusted to 2.5 with hydrochloride acid (1.0 mol/L). The volumes used of each solution are indicated in Table S2.

SIF-complete was prepared by mixing a duodenal juice and a bile solution. The stock solution used to make the duodenal juice consisted of sodium chloride (175.3 g/L), sodium bicarbonate (84.7 g/L), potassium dihydrogen phosphate (8.0 g/L), potassium chloride (89.6 g/L), magnesium chloride (5.0 g/L), urea (25.0 g/L), calcium chloride (22.2 g/L), bovine serum albumin (1.0 g/L), lipase (0.5 g/L), and pancreatin (3.0 g/L). The pH was adjusted to 7.5 with hydrochloride acid (1.0 mol/L). The bile solution was made with stock solutions of sodium chloride (175.3 g/L), sodium bicarbonate (84.7 g/L), potassium chloride (89.6 g/L), urea (25.0 g/L), calcium chloride (22.2 g/L), bovine serum albumin (1.7 g/L) and bile (40 g/L), which is equivalent to a bile concentration of ± 1%, as reported in the duodenum (Shah and Bergholz 2020). The pH was adjusted at 8.0 with sodium hydroxyde (1.0 mol/L). Three parts of duodenal solution and one part of the bile solution were mixed at room temperature to concoct the SIF-complete. The volumes of each solution are indicated in Table S2.

All the reagents and water were autoclaved for 15 min at 121 °C, and the enzyme solutions were prepared aseptically with sterile water and sterile filtered. The solutions were prepared and stored at 4 °C, except the enzyme solutions, which were made just before use. This protocol was previously published by Melo et al. (2013).

### Growth experiments in simulated gastrointestinal fluids

**Figure 1.**
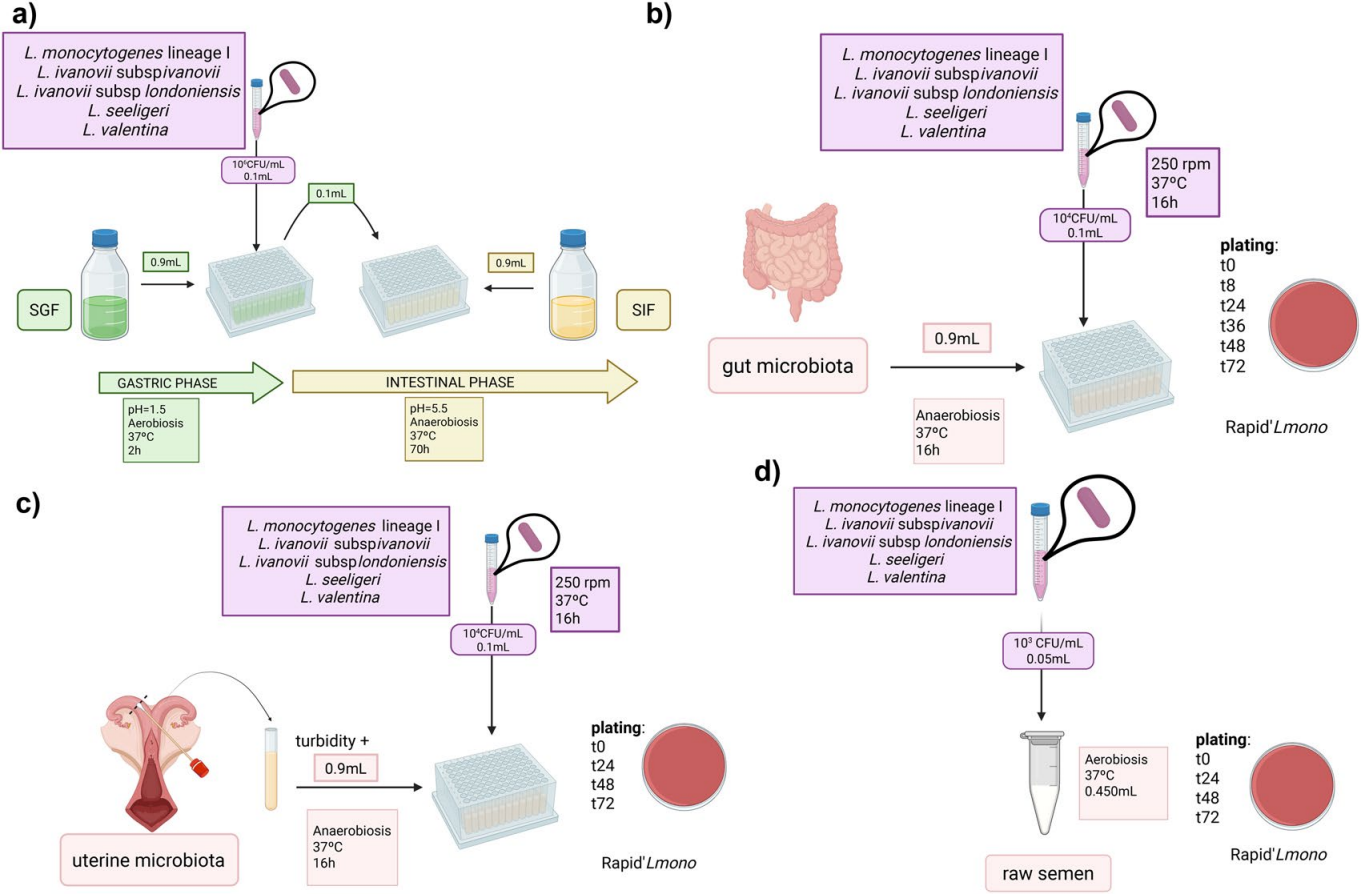
Experimental design for the study of *listeria* spp. growth abilities in a) SGF and SIF, b) an *ex vivo* gut microbiota, c) an *ex vivo* uterine microbiota, and d) raw semen.

The experimental process is adapted from Melo et al. (2013) and summarized in Figure 1a. Briefly, the overnight culture was adjusted to 10^6^ CFU/mL. Then 100 μL from the culture was mixed with 900 μL of SGF in a 96 deep-well plate, resulting in a final concentration of 10^5^ *Listeria* spp. CFU/mL. The inoculated SGF was then incubated for two hours at 37 °C in aerobiosis. After that, the culture was plated again, and 100 μL of the previously inoculated SGF was mixed with 900 μL of SIF-complete in a new deep-well plate and grown at 37 °C in anaerobiosis for an additional 70 h. Aliquots were withdrawn, diluted in PBS, and plated on BHI agar plates to enumerate the surviving *Listeria* at the subsequent times: 0, 2, 6, 10, 24, 48, and 72 h. The experiment was conducted, and the resulting data analyzed, between November and December 2024.

### Ethic statement

The sampling of the animals were approuved by the ethic of animal experimentation committee (CEEA: Comité de ética de experimentación animal) of the CEU Cardenal Herrera University. Moreover, the samplings were carried out on animals belonging to CEU Cardenal Herrera University during regulated practical classes held at the Faculty of Veterinary Medicine. The sampling of the animal is considered a routine academic and/or veterinary procedure; therefore, no further permit was required. For the same reason, no animal license numbers are available.

### Ex vivo gut model coculture experiments

The experimental process is schematized in Figure 1b. Fresh stools were collected from goats and processed within 1 h. The collected samples were pooled and treated as single biological replicates to minimize variation between individuals (Kauer et al. 2025). The goats used as donors in this study belonged to the farm of the CEU Cardenal Herrera University and shared housing, limiting the impact of a different environment on the microbiota. All the goats used were healthy, and none had shown listeriosis symptoms previously. The animals were kept and fed with their standard balanced diet, and fresh water was provided *ad libitum.* Animal handling and health measures were those recommended for this species under current legislation. The sample was collected on April 9, 2025, and the following experiment was made immediately after. Three g. of stool were mixed with 30 mL of reduced PBS and vortexed for 30 s. The supernatants were collected after stool sedimentation and mixed with AF medium (18 g/L brain-heart infusion (Oxoid), 15 g/L trypticase soy broth (Oxoid), 5 g/L yeast extract, 2.5 g/L K_2_HPO_4_, 1 mg/L hemin, 0.5 g./L D-glucose, 0.5 mg/L menadione, 3% heat-inactivated fetal calf serum, 0.25 g./L cysteine HCl‧H_2_O) supplemented with sodium carbonate (0.4 g/L) and mucin type II from porcine stomach (2.5 mg/L) at a 1:5 ratio (Weiss et al. 2022). The microbiota in AF broth was then incubated at 37 °C overnight in an anaerobic workstation (Whitley A25). The resulting *ex vivo* microbiota was used for the following experiment.

For the assessment of *Listeria* spp. survival, each strain was cultured overnight at 37 °C and 250 rpm. The OD of each strain was measured at 600 nm, and the overnight diluted 1:10 in PBS to reach 10^6^ CFU/mL. Then, 900 µL of *ex vivo* microbiota were inoculated with 100 µL of *Listeria* spp. overnight culture (*Listeria* final concentration: 10^5^ CFU/mL) in 96-deep well plates, one strain per column, following the method proposed by Li et al. (2019). After 0, 8, 20, 32, 48, and 72 h, six drops of 10 µL were plated in Rapid’*L.mono* agar plates (Bio-Rad) to assess *Listeria* spp. survival when cultured with an intestinal microbiota *ex vivo*. All the data was analyzed in the following month.

### Ex vivo uterine microbiota model coculture experiments

To explore the growth potential of *Listeria* species in the urogenital tract, we used a model replicating the uterine microbiota from an ewe. To do so, we sampled recently euthanised ewes culled for educational purposes from the authors’ university. The samples were collected in the first two weeks of May 2025 and processed immediately after (one sample was collected on May 1, and two samples on May 8). To euthanize the animals, an intravenous injection of barbiturate was used. Then, complete necropsies were carried out. Once the ewe was stabilized on the necropsy table, we checked for obvious reproductive lesions in the genital tract and for possible pregnancy. Only non-pregnant and without visible genital alterations ewes were selected for this study. We then disinfected the necessary material and one uterus horn with alcohol and opened a small window in its wall. We inserted one sterile standard calcium alginate swab (Puritan CalgiSwab) in the uterus horn, rubbing the uterine cavity firmly from the horn to the uterine body. The tip of the swab was cut to be introduced into a sterile Falcon tube filled with 30 mL of AF medium. The falcon tube was incubated at 37 °C in anaerobiosis for 16 h. The culture was considered successful if turbidity was observed the following day.

For *Listeria* spp. survival assessment, each strain was cultured overnight at 37 °C and 250 rpm. Overnight cultures were measured at 600 nm, diluted 1:10 in PBS 1x to reach 10^3^ CFU/mL. Then, 900 µL of *ex vivo* microbiota per well in 96-deep well plates were inoculated with 100 µL of *Listeria* spp. (*Listeria* final concentration: 10^2^ CFU/mL), one *Listeria* strain per column, following the method proposed by Li et al. (2019). After 0, 24, 48, and 72 h, six drops of 10 µL were plated in Rapid’*L.mono* agar plates (Bio-Rad) to assess *Listeria* spp. survival when cultured with a uterine microbiota *ex vivo*. This experimental process is resumed in Figure 1c. All the data was analyzed in the following month.

### Growth experiments in semen

We selected four adult and healthy rams with proven fertility belonging to the flock kept at the CEU Cardenal Herrera University farm. The animals were fed with their standard balanced diet, and fresh water was provided *ad libitum*. Animal handling and health measures were those recommended for this species under current legislation. Rams belonging to this ovine experimental herd have never shown symptoms of listeriosis before. The raw ejaculate samples were collected with an artificial vagina, brought back to the lab, and pooled (Bahadi et al. 2023). They were aliquoted in five sterile eppendorfs tubes and kept in the dark at 37 °C. As previously detailed, *Listeria* spp. overnight cultures were diluted and inoculated in the semen at an inoculum level of 10^2^ CFU/mL. At each time point (0, 24, 48, and 72 h), 6 drops of 10 µL were plated in Rapid’*L.mono* agar plates (Bio-Rad) to assess *Listeria* spp. survival, as shown in Figure 1d. The semen samples were extracted the 15^th^ of May 2025 and inoculated with *Listeria* spp. within the hour. All the data was analyzed in the following month.

### Statistical analysis

To examine the statistical differences between species, the analysis of variance test (ANOVA) and Kruskal-wallis tests were performed by using SPSS (IBM, version 27.01.0). The tests were applied to logarithmically transformed counts, calculated area under the curve (AUC), calculated growth rate, and cell survival percentages. In the SGF and SIF experiments, as well as.in the co-culture of *Listeria* with microbiota, 6 technical replicates of each isolate were made. For the validation of the culture of uterine microbiota (*Listeria* spp. in AF medium) and the inoculation of *Listeria* spp. in semen, 4 technical replicates were made. Differences were considered significant when *P* ≤ 0.05.

## Results

### Survival in simulated gastrointestinal fluids

**Figure 2.**
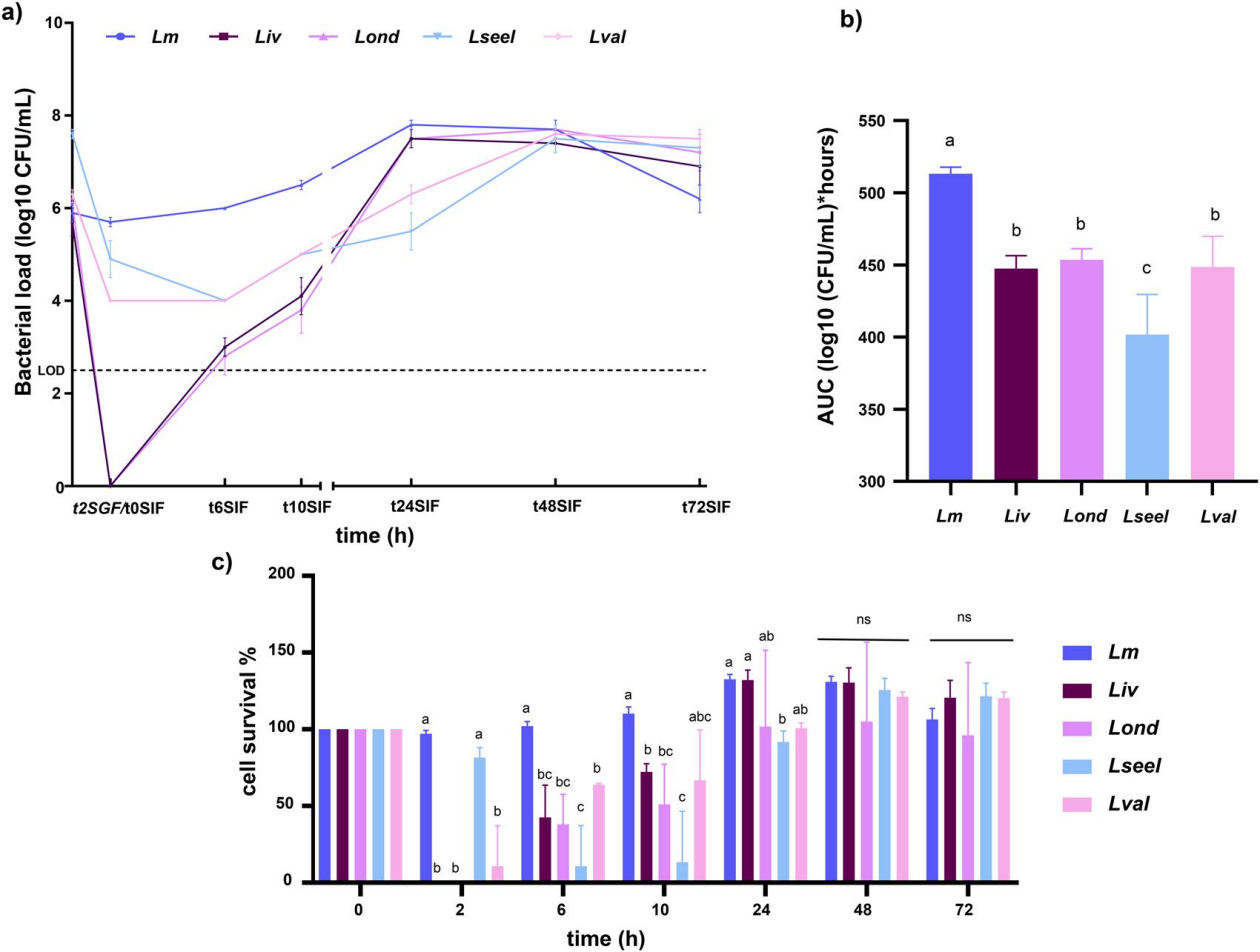
a) Growth curve, b) AUC, and c) % of cell survival of *Lm*, *Liv*, *Lond*, *Lseel*, and *Lval* in SGF and SIF (*n* = 6 replicates). Error bars represent the standard deviation. Different letters represent statistical differences (*P* < 0.05). ns stands for not significantly different.

The survival of *Listeria* species cells in simulated gastrointestinal conditions is illustrated in Figure 2. SGF exposure for two hours had variable inhibitory activity but reduced *Listeria* species viable CFU in all cases. The kinetics of SGF clearance varied significantly across the different *Listeria* species tested, indicating that acidic conditions of the digestive system do not contribute to the same extent to eliminating the *Listeria* species. As previously reported, a lack of substantial sublethal injury in *Lm* was detected (Barmpalia-Davis et al. 2008; Ramalheira et al. 2010; Rahman et al. 2020), probably due to acid adaptation mechanisms. Both clones of the subspecies of *L. ivanovii* displayed the highest acid sensitivity (*P* < 0.05), followed by *Lseel* and *Lval.* All the tested *Listeria* species recovered in the intestinal fluid (Figure 2a and c). *Lm* reached the stationary phase (7.8 Log_10_ CFU/ml) in SIF at 24 h (Figure 2a). Upon virtual clearance after SGF contact, SIF exposure resulted in a marked bloom of *Liv* and *Lond*. Thus, despite negative SGF cultures, residual *Liv* and *Lond* bacteria can undergo expansion in SIF when commensal microbes are absent. Even so, the AUC of *L. monocytogenes* was higher than *Liv* and *Lond* AUC in simulated gastrointestinal fluids (Figure 2b). *Lseel* and *Lval* demonstrated reduced growth in SGF and SIF compared to *L. monocytogenes* during the first 24 h. *Lseel* and *Lval* reached the stationary phase within 48 h of incubation in SIF medium at 37 °C and showed similar CFU counts to *Lm*, *Liv*, and *Lond*. Altogether, these data show that all the clones of the *Listeria* species tested grew exponentially in SIF without commensal bacteria, indicating that neither bile acids nor pancreatic enzymes provide bactericidal effects.

### Ex vivo competition with gut microbiota

**Figure 3.**
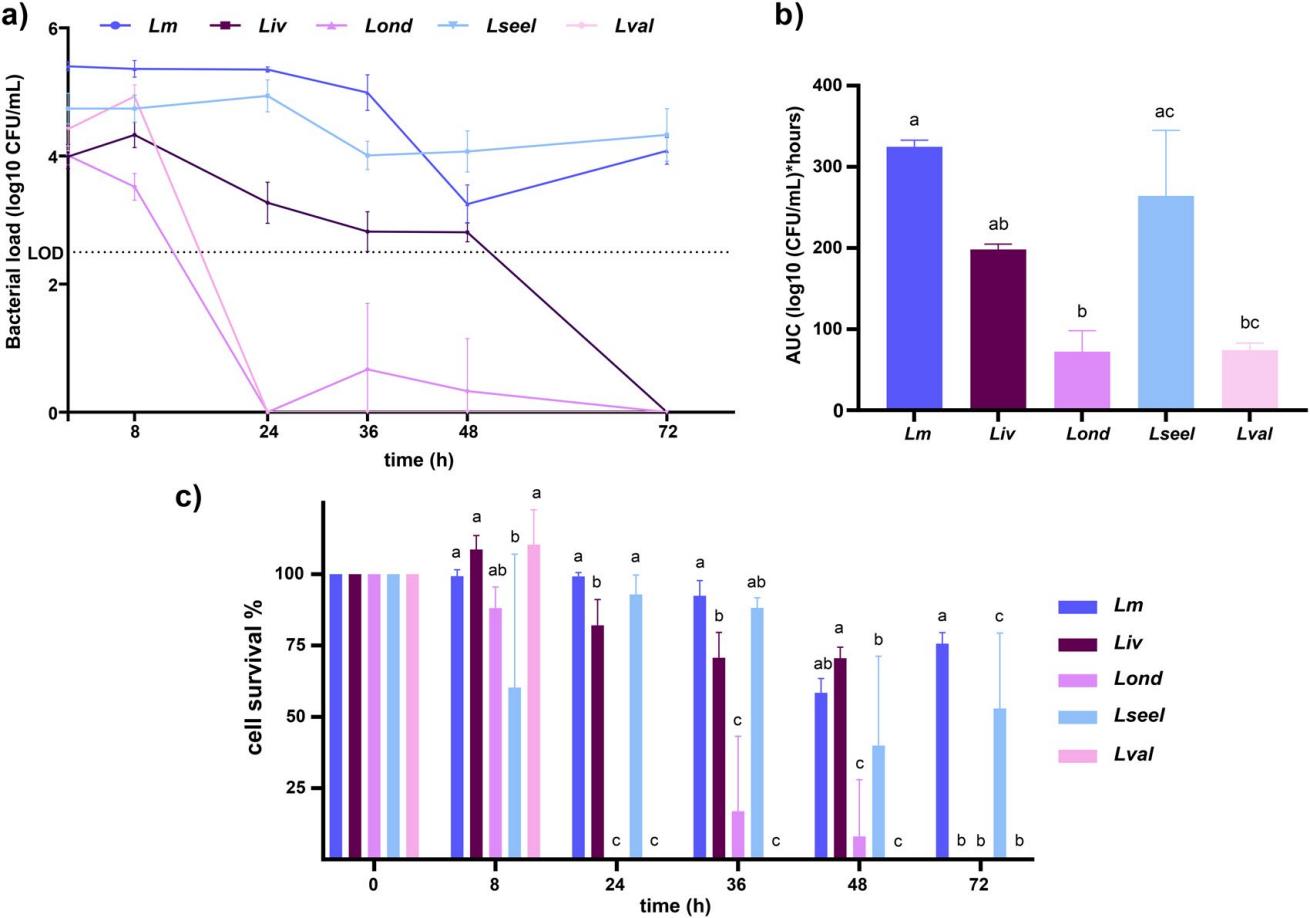
a) Growth curve, b) AUC, and c) % of cell survival of *Lm*, *Liv*, *Lond*, *Lseel,* and *Lval* in gut microbiota *ex vivo* (*n* = 6 replicates). Error bars represent the standard deviation. Different letters represent statistical differences (*P*< 0.05).

To investigate the ruminant gut microbiota resistance against oral *Listeria* species infection, we used an *ex vivo* model, which has been previously shown to be a good model for intestinal infection (Becattini et al. 2017). *Ex vivo* experiments showed that commensals mediated *Listeria* species clearance at different proportions (Figure 3a–c). Gut microbiota inhibited *Lm* and *Lseel* CFUs by over two orders of magnitude within 72 h, reaching a final concentration of 4 Log_10_ CFU/mL (Figure 3a). In contrast, *Lond* and *Lval* were rendered undetectable by the gut microbiota within 24 h, and *Liv* within 72 h. Altogether, these results showed that gut commensals displayed less inhibitory activity against the tested isolate of *Lm* than against the isolates of *Liv* and *Lond*, although in all cases, they reduced viable CFUs over 72 h.

### Ex vivo competition with uterine microbiota

**Figure 4.**
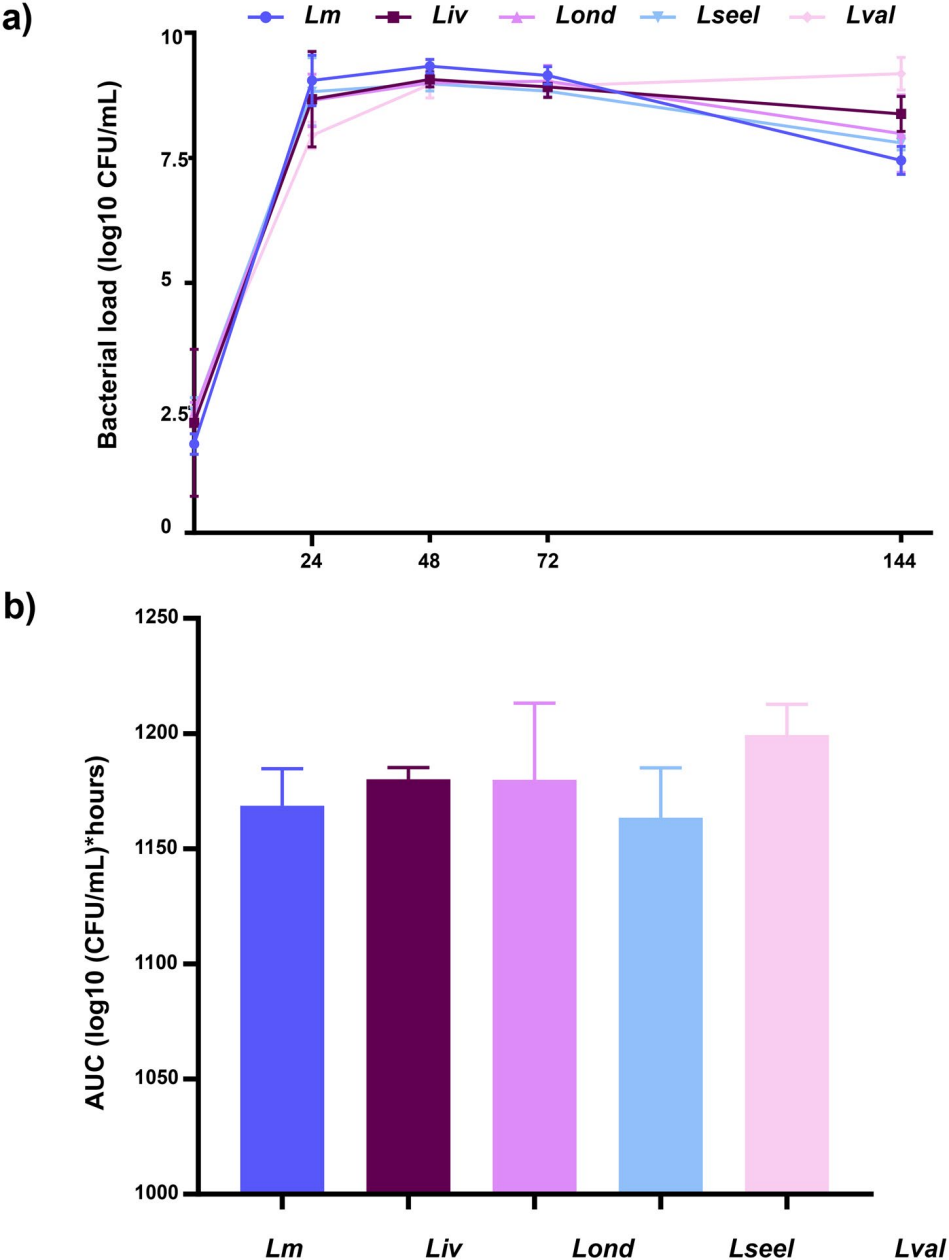
a) Growth curve and b) AUC of *Lm*, *Liv*, *Lond*, *Lseel,* and *Lval* in AF medium (*n* = 6 replicates). Error bars indicate the standard deviation.

**Figure 5.**
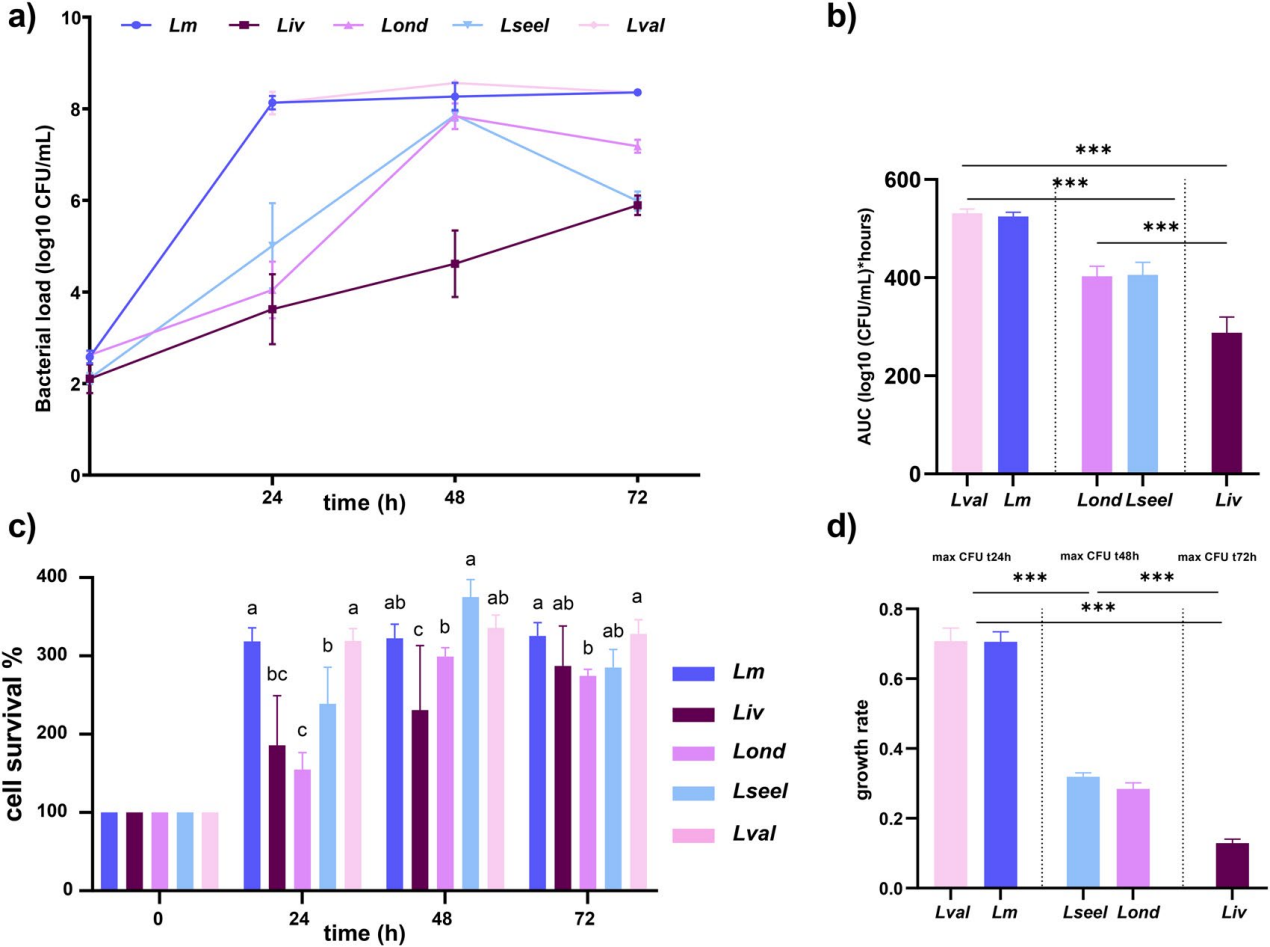
a) Growth curve, b) AUC, c) cell survival percentage, and d) growth rate of *Lm*, *Liv*, *Lond*, *Lseel,* and *Lval* in uterine microbiota *ex vivo* (*n* = 6 replicates). Error bars represent the standard deviation. In figures 5b and d, statistical differences are represented by three stars (P< 0.001). In Figure 5c), different letters represent statistical differences ( *P*< 0.05).

As the literature concerning the culture of uterine microbiota from ruminants is lacking, we first validated the model used in this study. We successfully cultivated the ewe’s uterine microbiota to 9.0 and 11.8 Log_10_ CFU/mL (Supplementary Figure 1). We then compared the growth of *Listeria* species in AF medium with and without microbiota to evaluate whether the potential differences observed between species could be attributed to their specific ability in competing against the uterine microbiota (Figures 4a and b and 5a–d). In AF medium, all the strains reached maximum counts in the first 24h, with no difference between them. At the end of the experiment at 144 h, no statistical differences were found between the five *Listeria* species when grown in AF medium without microbiota (Figure 4a and b).

Next, we cultivated the same five *Listeria* species in AF medium with uterine microbiota and observed different growth patterns (Figure 5d). As the only difference between these two experimental setups is the presence or absence of the microbiota, these results suggest that the presence of uterine commensals influences the growth of our five strains. Uterine microbiota restricted the growth of *Listeria* species compared to the AF medium alone (Figures 4a and b and 5a–d). In contact with uterine microbiota, nonpathogenic *Lval* and pathogenic *Lm* reached higher counts than the other species, reaching their highest concentration in 24 h (Figure 5a, c, and d). Although *Lond* and *Lseel* growth was slower, they reached similar values to *Lm* and *Lval* after 48h. Finally, although *Liv* presented the slowest growth, it was able to survive, multiply, and finally reach a concentration of 5.9 Log_10_ CFU/ml at 72 h (Figure 5a). In contrast with the previous results where the tested isolates of *Liv*, *Lond*, and *Lval* could not survive to gut microbiota exposure, here all could compete with the uterine microbiota and grow.

### *Listeria* species growth in semen

**Figure 6.**
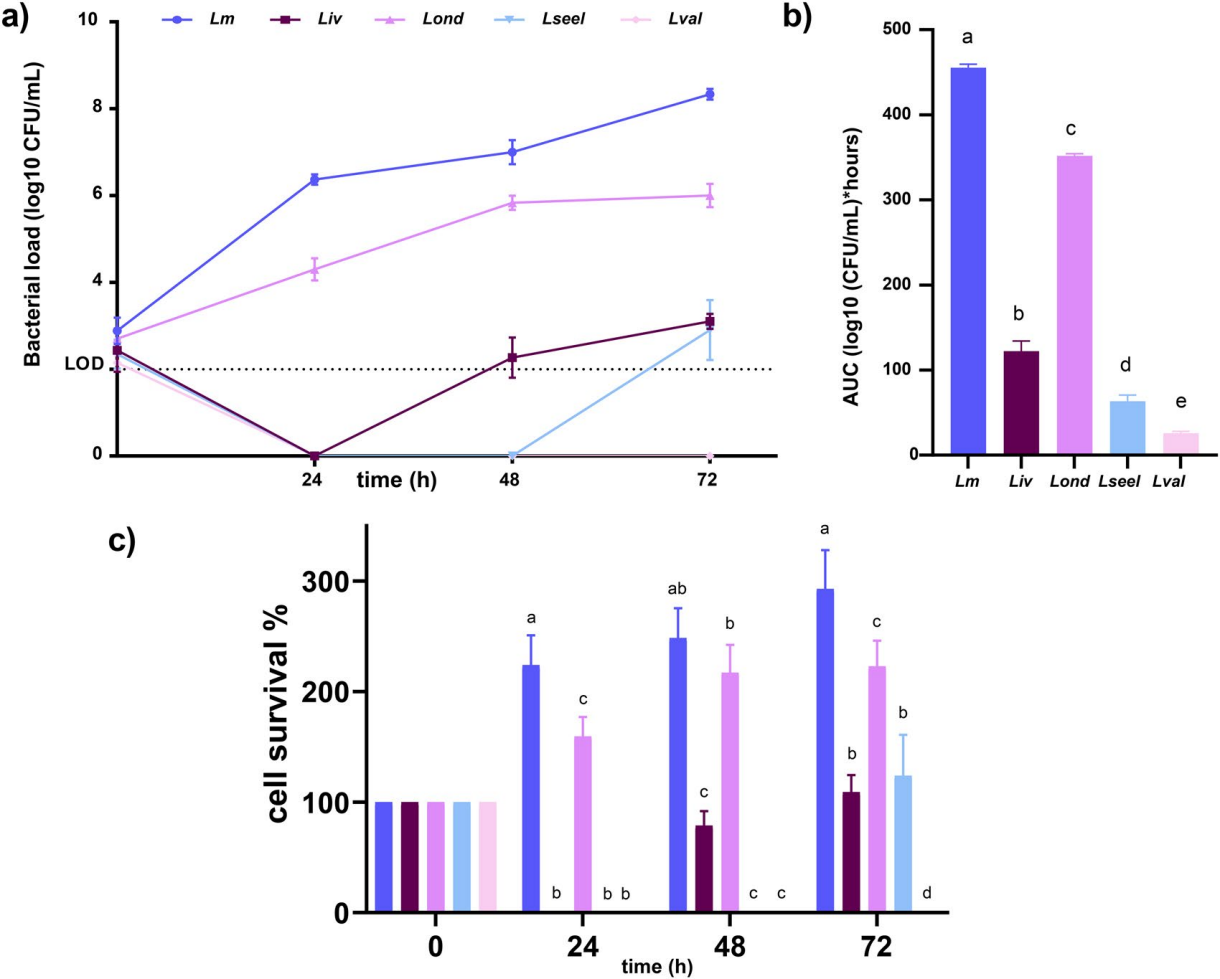
a) Growth curve, b) AUC, and c) cell survival percentage of *Lm*, *Liv*, *Lond*, *Lseel,* and *Lval* grown in semen at 37 °C (*n* = 4 replicates). Error bars represent the standard deviation. Different letters represent a significant difference (*P* < 0.05).

The survival and growth of *Listeria* species cells in semen are illustrated in Figure 6. Semen exposure had variable inhibitory activity. *Lval* could not replicate in this environment and was undetectable during the experiment. *Liv* and *Lseel* CFUs decreased in the first 24 h and 48 h, respectively, although they finally grew to 10^3^ CFU/mL after 72 h. *Lm* and *Lond* constantly grew up to 72 h and expanded to 8.3 and 6 Log_10_ CFU/ml, respectively. Altogether, these data show that the isolates of pathogenic *Listeria* species can grow in semen.

## Discussion

Studies about the ecological preferences of different *Listeria* species are necessary to understand their distribution in nature. Although *Lm* and *Liv/Lond* are pathogenic species, two remarkable differences exist between them: i) while *Lm* causes septicemia, encephalitis, and reproductive disorders in humans and ruminants, *Liv/Lond* only causes reproductive disorders in ruminants (Sergeant et al. 1991; Alexander et al. 1992; Gill et al. 1997; Vázquez-Boland et al. 2001); ii) *Lm* is significantly more abundant than *Liv/Lond* in hosts, soils and food (MacGowan et al. 1994; Hafner et al. 2021; Palacios-Gorba et al. 2021a). The present study helps to understand why *Lm and Liv/Lond* have distinct host niche preferences. Our results and previous studies suggest that establishing an asymptomatic fecal carriage plays a fundamental role in *Listeria* spp. ecology since survival to gastrointestinal conditions may favor host intestinal colonization, replication, and fecal shedding. Fecal excretion finally helps *Listeria* to transit between the host intestine, soil, and food (Nightingale et al. 2004; Hafner et al. 2021; Palacios-Gorba et al. 2021a). Extrapolating the present data to a species level, *Lm* and *Lseel* resistance against gastrointestinal fluids, as well as competitiveness against gut microbiota, appear to ensure their higher distribution in urban, ruminant farm, and natural environments than *L. ivanovii* (MacGowan et al. 1994; Sauders et al. 2012; Chapin et al. 2014; Linke et al. 2014; Orsi and Wiedmann 2016; Palacios-Gorba et al. 2021a; Liao et al. 2023). In contrast, our results demonstrate that bacteria inhabiting the intestine allow the inhibition of the tested clones of *Liv* and *Lond* growth. Possible mechanisms may include nutrient competition (Maltby et al. 2013), production of antibacterial molecules (Zhu et al. 2000; Corr et al. 2007; Lakshminarayanan et al. 2013; Vijayakumar and Muriana 2015; Egan et al. 2016; Saraoui et al. 2016), as well as contact-dependent inhibition (Ruhe et al. 2013). Altogether, presuming that the behavior of the tested isolates is representative of the species, our results show that acidic conditions of the digestive system and the intestinal microbiota provide the first-line defense against orally acquired *Liv* and *Lond* infection, preventing gut colonization, replication, and fecal shedding. The different environmental distribution of *Lm* versus *Liv*/*Lond* could also be due to differences in environmental persistence, transmission dynamics and host immune evasion. These latter hypotheses reserve further investigation.

On the contrary, the growth of *Lond* -and at a lower rate, *Liv* - in contact with the uterine microbiota indicates that the female reproductive tract may be a more permissive niche than the gastrointestinal tract. This niche preference could explain why *Liv/Lond* are associated with abortion outbreaks in ruminants (Macleod et al. 1974; Dennis 1975; Sergeant et al. 1991; Alexander et al. 1992; Gill et al. 1997; Chand and Sadana 1999; Şahi̇n and Beytut 2006).

The hypothesis of sexual transmission for *Listeria* has been previously discussed by other authors in humans as well as in other mammals (Osebold and Inouye 1954; Rappaport et al. 1960; Toaff et al. 1962; Gray 1963; Gray and Killinger 1966; McDonald 1967; Smith et al. 1967; Wiedmann et al. 1999). This hypothesis was based on the isolation of pathogenic *Listeria* from the genital tract of healthy humans and animals, as well as during abortion cases. Documented cases, such as the one described by Toaff et al. Rappaport et al. and Gray’s experiments in the 60’s showed the chronic presence of *Listeria* in the uterus, cervix, or vagina of aborted women and does, and in the semen of their interacting males. *L. ivanovii* (also called *Lm* serotype 5) was also suspected to be of venereal transmission in some sheep’s abortion cases (McDonald 1967; Smith et al. 1967; Alexander et al. 1992). As proposed by Lennon et al. (1984), more investigation on the possible venereal transmission of *L. ivanovii* and *Lm* should be done, especially considering that MacPherson and Fish (1954) demonstrated that pathogenic *Listeria* could survive in frozen bovine semen.

In conclusion, the present study contributes to the understanding of the different *Listeria* species’ preferences for distinct ecological niches and clinical manifestations of *L. monocytogenes* and *L. ivanovii*.

## Supplementary Material

Supplementary Table 2.pdf

CEEA 25_17_QUEREDA_Informe CEEA.pdf

## Data Availability

The data that support the findings of this study are openly available in a data repository (www.Figshare.com) with the following title: Tissue-specific microbiota dictates the competitive dynamics of Listeria species colonization (Version 2). figshare and can be accessed online at: https://doi.org/10.6084/m9.figshare.30075817.v2 ((Poujol de Molliens 2025).
