# Supplementary material for: Tissue-specific microbiota dictates the competitive dynamics of listeria species colonization
Source: Vet Q. 2026 Feb 2;46(1):2622742. doi: 10.1080/01652176.2026.2622742 (PMC12865829; doi:10.1080/01652176.2026.2622742)
Supplement: Supplementary Table 2.pdf [file TVEQ_A_2622742_SM7482.pdf]

preparation SGF 50mL			
	inorganic solution mL		organic solution mL
NaCl	0,785	glucose	0,5
NaH2PO4	0,15	glucuronic acid	0,5
KCl	0,46	urea	0,17
CaCl2	0,9	glucosamine hydrochloride	0,5
NH4Cl	0,5	water	23,3
HCl	0,415	total	25
water	21,8		
total	25		
	mix the two solutions and add (g)		
	BSA	0,05	
	Pepsin	0,05	
	Mucin	0,15	

preparation SIF 500mL					
duodenal juice 500mL			bile solution 150mL		
	inorganic solution mL	organic solution mL		inorganic solution mL	organic solution mL
KCl	3,15		NaCl	4,5	
NaCl	20		$NaHCO_3$	10,245	
$NaHCO_3$	20		KCl	0,63	
KH_2PO_4	5		HCl	0,03	
$MgCl_2$	5		urea		1,5
$CaCl_2$	4,5		agua	59,7	73,5
HCl	0,09		total	75	75
urea		2	mix both solution and add (g)		
agua	192,3	248		$CaCl_2$	1,5
total	250	250		BSA	0,15
	mix both solution and add (g)				
	$CaCl_2$	4,5			
	pancreatine	1,5			
	lipase	0,25			
	BSA	0,5			
mix 3 parts of duodenal juice with 1 part of bile solution (375mL+125mL=500mL) and add 16g of bile (1%)					

Table S2: SGF and SIF composition and volumes of stock solutions used (mL).
