# Supplementary material for: Tissue-specific microbiota dictates the competitive dynamics of listeria species colonization
Source: Vet Q. 2026 Feb 2;46(1):2622742. doi: 10.1080/01652176.2026.2622742 (PMC12865829; doi:10.1080/01652176.2026.2622742)
Supplement: CEEA 25_17_QUEREDA_Informe CEEA.pdf [file TVEQ_A_2622742_SM7481.pdf]

INFORME CEEA 25/17

El Comité de Ética de Experimentación Animal (CEEA), reunido con fecha 31 de marzo de 2025 ha revisado el proyecto con título «**Estudio del crecimiento de cepas de *listeria spp*. en una modelización del aparato genital de la especie *Ovis aries***» remitido por el investigador Juan José Quereda Torres

Según el artículo 2.5. del Real Decreto 53/2013 del 1 de febrero, por el que se establecen las normas básicas aplicables para la protección de los animales utilizados en experimentación y otros fines científicos, incluyendo la docencia (BOE 8.02.2013), especifica que quedan excluidas del ámbito de su aplicación:

a) Las prácticas agropecuarias no experimentales;
b) Las prácticas veterinarias clínicas no experimentales;
c) Los estudios veterinarios clínicos necesarios en el marco de la obtención de la autorización de comercialización de medicamentos veterinarios;
d) Las prácticas realizadas con fines zootécnicos reconocidos;
e) Las prácticas realizadas con el objetivo principal de identificar un animal;
f) Las prácticas en las que no sea probable que se les ocasione dolor, sufrimiento, angustia o daño duradero equivalentes o superiores a los causados por la introducción de una aguja conforme a las buenas prácticas veterinarias.

Por lo tanto, a la vista de la descripción de este proyecto, no entra en el ámbito de aplicación del mencionado RD.

Alfara del Patriarca, 2 de mayo de 2025

LA PRESIDENTA

Fdo. Alicia López Castellano
